# *Angiostrongylus cantonensis* causes cognitive impairments in heavily infected BALB/c and C57BL/6 mice

**DOI:** 10.1186/s13071-020-04230-y

**Published:** 2020-08-10

**Authors:** Kai-Yuan Jhan, Guan-Jhih Lai, Pi-Kai Chang, Ren-Yu Tang, Chien-Ju Cheng, Kuang-Yao Chen, Lian-Chen Wang

**Affiliations:** 1 Department of Parasitology, College of Medicine, Chang Gung University, Taoyuan, 333 Taiwan; 2 Graduate Institute of Biomedical Sciences, College of Medicine, Chang Gung University, Taoyuan, 333 Taiwan; 3 Molecular Infectious Disease Research Center, Chang Gung Memorial Hospital, Taoyuan, Taiwan

**Keywords:** *Angiostrongylus cantonensis*, Behavior, Forced swimming test, Learning, Memory, Morris water maze test, Open field test

## Abstract

**Background:**

Parasitic infections may cause significant effects on behavior, learning, and memory of the host. In the brain of mice heavily infected with *Angiostrongylus cantonensis*, severe damage has been observed in the hippocampus. This component has been considered to have associations with spatial learning and memory in humans and vertebrates. This study was designed to determine the impairments in behavior, learning, and memory in BALB/c and C57BL/6 mice heavily infected with the parasite.

**Methods:**

Each mouse was inoculated with 50 third-stage larvae of *A*. *cantonensis*. After infection, daily changes in weight and dietary consumption, worm recoveries and survival rates were determined. The forced swimming test, open field test, and Morris water maze test were employed to evaluate depression- and anxiety-like behavior as well as impairments in spatial learning and memory, respectively.

**Results:**

The worm recovery rate in the BALB/c mice was significantly lower than that of C57BL/6 mice from day 14 post-infection. The survival rate in infected BALB/c mice decreased to 0% by day 25 whereas those with swim-training survived three more days. On day 42, the C57BL/6 mice had a survival rate of 85.7% in the swimming group and 70% in the non-swimming group. Significant differences were found in weight between infected and non-infected BALB/c and C57BL/6 mice from day 13 and day 12, respectively with corresponding changes in their dietary consumption. Depression-like behavior was found in the infected BALB/c mice but not in C57BL/6 mice. However, anxiety-like behavior was found to occur only in C57BL/6 mice. Impaired spatial learning and memory were also found in the two strains of mice which occurred from day 14 post-infection.

**Conclusions:**

Results of this study indicate that *A*. *cantonensis* causes depression, anxiety, and impairments in spatial learning and memory in heavily infected mice. Moreover, significantly higher severity was observed in the Th-2 dominant BALB/c mice.
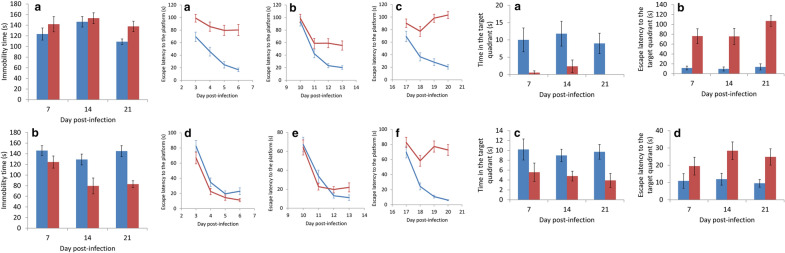

## Background

Parasitic infections have been reported to have significant effects on behavior, learning, and memory of the host. Mice infected with *Heligmosomoides polygyrus* and *Eimeria vermiformis* were respectively observed to have poor spatial learning and a reduced avoidance of cat odor [1, 2]. However, rodents heavily infected with *Strongyloides ratti* did not show deficits in learning [3]. Although inconsistent findings were obtained in infected rodents with gastrointestinal parasites, neurotropic parasites such as *Toxocara canis* [4] and *Toxoplasma gondii* [5–7] have been documented to cause learning impairments in their hosts. Moreover, the presence of cysts of *T*. *gondii* in specific forebrain areas of rats has been attributed to attenuation of predator odor aversion and changes in anxiety-related behavior [6]. Association between *T*. *gondii* and anxiety and cognitive disorders has also been reported [8, 9].

*Angiostrongylus cantonensis* is a neurotropic nematode causing eosinophilic meningitis and/or eosinophilic meningoencephalitis in humans. The infection is due to ingestion of raw or uncooked intermediate hosts (snails or slugs), paratenic hosts (crustaceans, frogs, fishes or planarians), or vegetables contaminated with the infective third-stage larvae (L3) [10, 11]. After ingestion, L3 penetrate the small intestine and reach the central nervous system through the bloodstream. In the brain, the worms molt twice and develop into the fifth-stage larvae or young adults. The young adults cause a series of pathological and immunological changes [12]. Severe headaches, fever, nausea, vomiting, neck stiffness, and neurologic abnormalities are the common clinical manifestations persisting for weeks to months. Although most human infections are self-limited, severe infection may lead to irreversible neurological damages and even death [13]. Using the water maze, BALB/c mice have been shown to have learning and memory deficits 1–2 weeks post-infection [14].

In our previous studies, we have reported that *A. cantonensis* causes significant pathological changes in the brain of infected mice especially in the hippocampus [15] and different temporal-spatial expressions of IL-4, IL-10, and IL-13 between of the infected Th-2 dominant BALB/c and Th-1 dominant C57BL/6 mice [16]. Rats with hippocampal damage required a prolonged duration to find the test platform in the Morris water test [17]. Moreover, the hippocampal gyrus has been related to depression [18, 19]. Daily feeding of *Lactobacillus helveticus* to rats with ammonium acetate induced chronic hyperammonemia has been reported to elevate cognitive functions [20]. Treatment with the emotional stabilizer LiCl to young rats inoculated with *Streptococcus pneumoniae* not only prevented apoptosis and reduced damages in hippocampus, but also improved spatial memory and learning [21]. In addition, icariin has been effective in treating mice with traumatic brain injuries [22]. Since *A. cantonensis* infection causes damage to the hippocampus, these changes may lead to impairments in the behavior, learning, and memory of the host. In this study, we employed the force swimming test to evaluate the level of depression, the open field test to assess anxiety, and the Morris water maze test to determine changes in learning and memory in heavily infected BALB/c and C57BL/6 mice with *A. cantonensis*.

## Methods

### Parasite and laboratory animals

A Taiwan strain of *A*. *cantonensis* has been maintained in our laboratory since 1980. Its life-cycle is maintained through *Biomphalaria glabrata* snails and Sprague-Dawley (SD) rats [16]. The rats were used for life-cycle maintenance and 7–8-week-old BALB/c (H-2^d^) and C57BL/6 (H-2^b^) mice were used for experimental studies. Animals were purchased from the National Laboratory Animal Center (Taipei, Taiwan) and BioLASCO Taiwan Co., Ltd. (Taipei, Taiwan). These animals were reared in the Laboratory Animal Center of Chang-Gung University. They were kept in plastic cages and provided with food and water *ad libitum*. All procedures were reviewed and approved by the Institutional Animal Care and Use Committee of Chang Gung University (IACUC Approval No.: CGU15-193).

### Experimental infection

On day 21 post-infection, tissues of the snail hosts were removed and homogenized after shell crushing. The tissue samples were digested with 0.6% (w/v) pepsin-HCl (pH 2–3) for at 37 °C for 1 h [23]. L3 of *A*. *cantonensis* were removed from the standing precipitations using a medical dropper and counted under a dissecting microscope. Each mouse was inoculated with 50 L3 by stomach intubation.

### Determination of weight and dietary consumption

Mice undertaking the tests have to exercise in the training and testing stages, as it was a requirement to determine whether exercise affected the status of infection. There were two controls groups (10 BALB/c mice and 10 C57BL/6 mice) and two corresponding infected groups with equal numbers. Throughout the experimental studies, each mouse was weighed daily. In addition, the daily dietary consumption was determined by recording the intake of food and drinking water.

### Forced swimming test

The forced swim test is a behavioral test used for the evaluation on the level of depression after *A*. *cantonensis* infection. There were 10 mice in each of the control and infected groups. The test was carried out on days 7, 14 and 21 post-infection. The tested mouse is placed in a transparent cylindrical tank (45 cm height × 10 cm radius) and filled with water (25 ± 1 °C) at a level of 15 cm from the bottom for 6 min. The duration that the mouse does not swim in the last 4 min is recorded [24].

### Open field test

The open field test was used to assess anxiety-like behavior in *A*. *cantonensis* infected mice. There were 10 mice in each of the control and infected groups. The test was carried out on day 9 post-infection. The open field apparatus consisted of a square tank (40 × 40 cm) with walls 40 cm high made of gray polyvinyl chloride plastic board. Mice were transported to the testing room and left in their home cages for 1 h. The animal was placed in the middle of the tank and allowed to move freely. Speed and time of the locomotor activity was monitored for 1 h. Tendency to anxiety was defined as time in the middle less than time on the edge [25].

### Morris water maze test

The Morris water maze test was used to assess the effect of *A*. *cantonensis* on spatial learning and memory. There were 10 mice in each of the control and infected groups. The mice were trained on days 3–6, 10–13 and 17–20 post-infection, and tests performed on days 7, 14 and 21 days post-infection. The custom-made maze was a circular pool 120 cm in diameter and 40 cm in height, filled with water at 25 ± 1 °C and made opaque by adding milk. In the acquisition session, each mouse was given 4 trials per day and 4 days of training in total to find a hidden platform located 1.5 cm below the water surface. Each mouse was placed into the pool, facing the wall, with a different starting point for each trial that the direct route to the platform differed each time. The time required by the mouse to find and stand on the platform was recorded for up to 90 s. The mouse was allowed to stay on the platform for 30 s. It was then removed from the maze and placed into its cage. For the mouse that found the platform within 90 s, the animal was placed on the platform for 30 s. The inter-trial interval was at least 30 min. In the probe session, on day 5, the platform was removed from the pool. The mouse was tested in a probe trial for 60 s. Mouse swimming tracts were recorded using a TopScan automated tracking system (Clever Sys Inc., Virginia, USA) [26].

### Determination of worm recovery

The infected mice were sacrificed by inhalation of 3% (v/v) isoflurane (Panion & BF Biotech Inc., Taipei, Taiwan). Brains were removed from the cranial cavity. Worm recovery rates were then determined by counting the number worms under a dissecting microscope.

### Statistical analyses

Statistical analyses were performed using Microsoft Excel 2010 for Windows (Microsoft, Redmond, WA, USA). Data were expressed as the mean ± standard deviation (SD). Differences between groups were analyzed by Student’s t-test. *P* < 0.05 was considered to be statistically significant.

## Results

### Worm recovery and survival rate

**Fig. 1:**
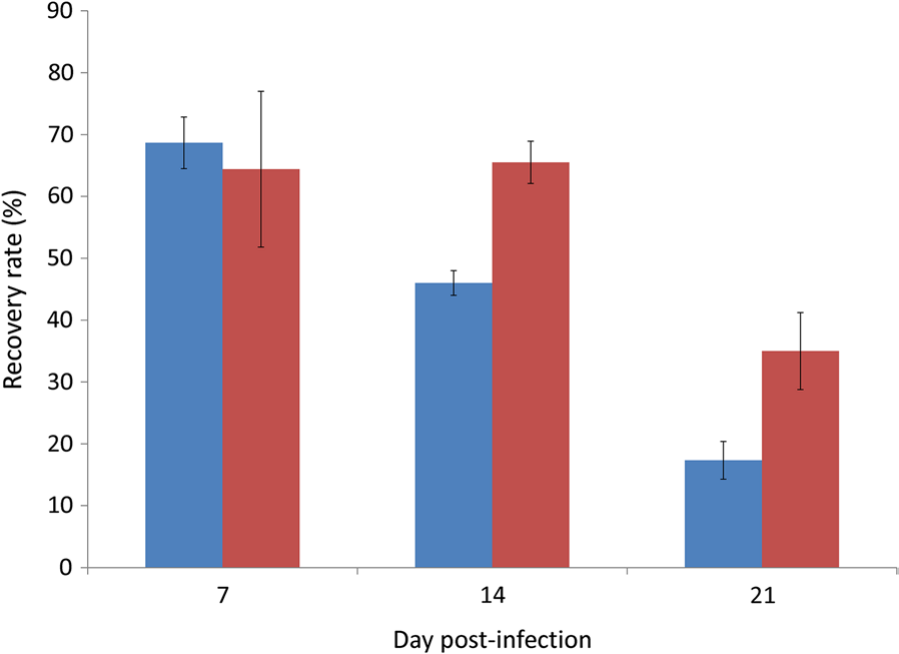
Comparison of worm recovery rates between BALB/c (blue) and C57BL/6 (red) mice infected with *Angiostrongylus cantonensis* and tested with the Morris water maze

The worm recovery rate in BALB/c mice tested with the Morris water maze significantly decreased with the time of infection (*t*_(4)_ = 0.000169, *P* < 0.001) whereas that of C57BL/6 mice decreased only on day 21 post-infection (*t*_(4)_ = 0.001452, *P* < 0.01). In addition, the rate in the BALB/c mice was significantly lower than that of C57BL/6 mice on days 14 (*t*_(4)_ = 0.000331, *P* < 0.001) and 21 (*t*_(4)_ = 0.006658, *P* < 0.01) (Fig. 1).

**Fig. 2:**
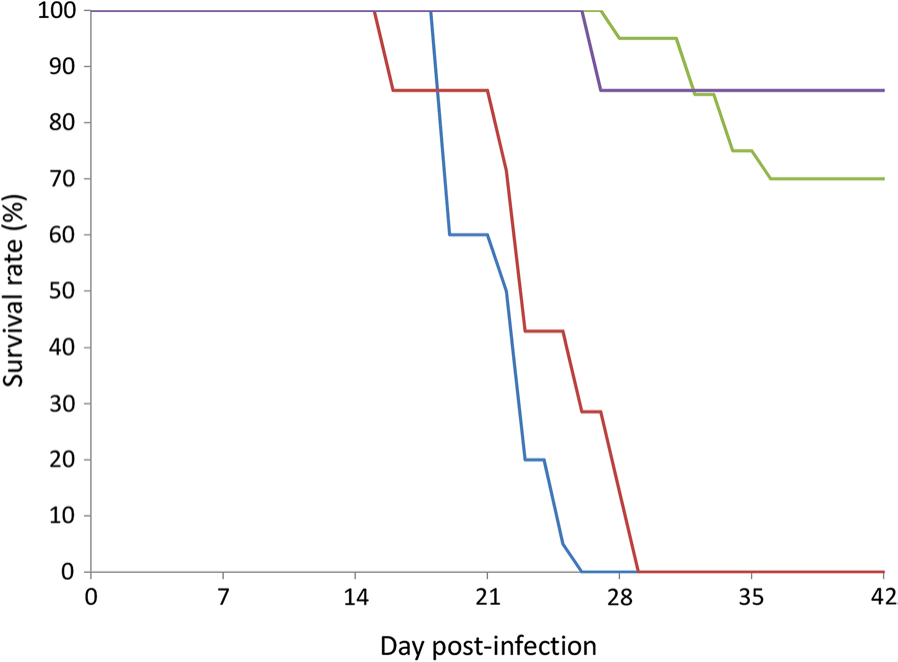
Effects of the Morris water maze test on the survival rates in BALB/c mice (blue, non-swimming group; red, swimming group) and C57BL/6 mice (purple, non-swimming group; green, swimming group) infected with *Angiostrongylus cantonensis*

All animals in the control groups remained alive on day 42 post-infection, giving a survival rate of 100% (data not shown). The survival rate in BALB/c mice without undertaking the water maze test (the non-swimming group) rapidly decreased to 0% by day 25 whereas those undertaking the test (the swimming group) survived three more days. Among the C57BL/6 mice, the survival rate only became 70% on day 42 in the non-swimming group and that in the swimming group remained 85.7% on the same day. The mortality rate in the swimming group decreased from 30% to 14% (Fig. 2).

### Changes in weight

**Fig. 3:**
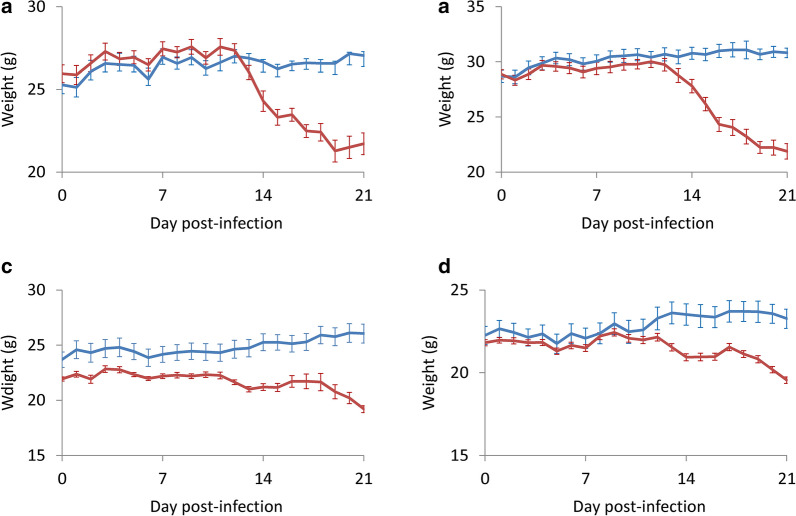
Effects of the Morris water maze test on the weight in BALB/c and C57BL/6 mice infected with *Angiostrongylus cantonensis*. **a** BALB/c non-swimming group. **b** BALB/c swimming group. **c** C57BL/6 non-swimming group. **d** C57BL/6 swimming group. *Key*: blue, uninfected; red, infected

In the non-swimming group, there was no significant difference in weight of infected and non-infected BALB/c mice up to day 13 post-infection (*t*_(9)_ = 0.320876, *P* > 0.05). From day 14, the weight of the infected group significantly decreased (*t*_(9)_ = 0.006903, *P* < 0.01) (Fig. 3a). On day 21, the non-infected mice had a weight gain of 7% and the infected mice had a weight loss of 24%. A similar decreasing pattern was observed in the swimming group. Significant differences in weight of the infected animals were found from day 13 (*t*_(9)_ = 0.011617, *P* < 0.05) (Fig. 3b). On day 21, the non-infected mice had a weight gain of 7% and the infected mice had a weight loss of 21%.

In the non-swimming group, the weights of infected C57BL/6 mice were found to be significantly lower than the controls (*t*_(9)_ = 0.031012, *P* < 0.05) (Fig. 3c). The infected mice had a weight loss of 12% on day 21 post-infection whereas the uninfected controls had a weight gain of 10%. In the swimming group, the C57BL/6 mice had a change pattern similar to their BALB/c counterparts. From days 1–11, no significant difference was found in the infected and uninfected mice (*t*_(9)_ = 0.326197, *P* > 0.05) and the weight of the infected mice significantly decreased from day 12 (*t*_(9)_ = 0.037645, *P* < 0.05) (Fig. 3d). On day 21, the uninfected mice also had a 10% weight gain while the infected mice only had a weight loss of 10%.

### Dietary consumption

**Fig. 4:**
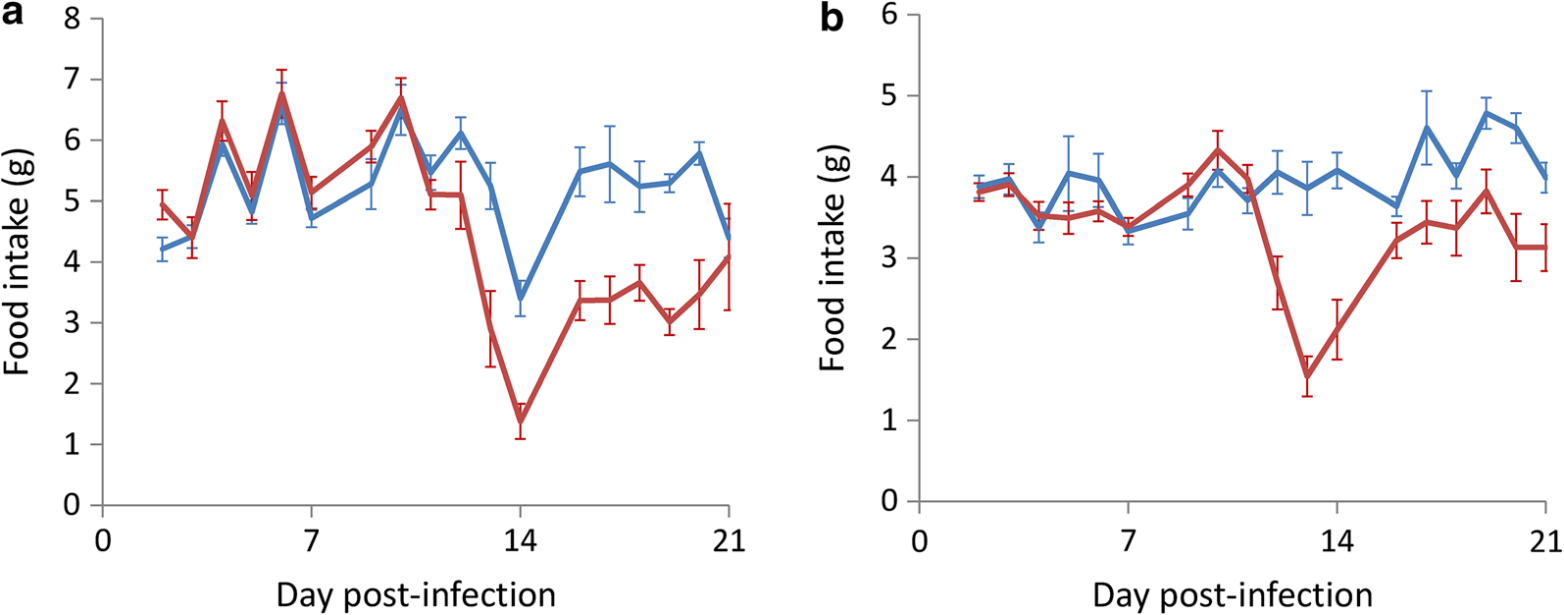
Effects of the Morris water maze test on the food intake in BALB/c (**a**) and C57BL/6 (**b**) mice infected with *Angiostrongylus cantonensis*. *Key*: blue, uninfected; red, infected

In BALB/c mice, there was no significant difference in food intake between the uninfected control and infected mice in the swimming group up to day 12 post-infection (*t*_(9)_ = 0.113181, *P* > 0.05). From day 13, food intake in the infected group significantly decreased (*t*_(9)_ = 0.004678, *P* < 0.01) (Fig. 4a). There was also no difference in food intake in C57BL/6 infected mice in the swimming group and the uninfected controls up to day 12 (*t*_(9)_ = 0.692658, *P* > 0.05). However, food intake in the infected mice significantly decreased from day 13 (*t*_(9)_ = 0.004806, *P* < 0.01) (Fig. 4b).

**Fig. 5:**
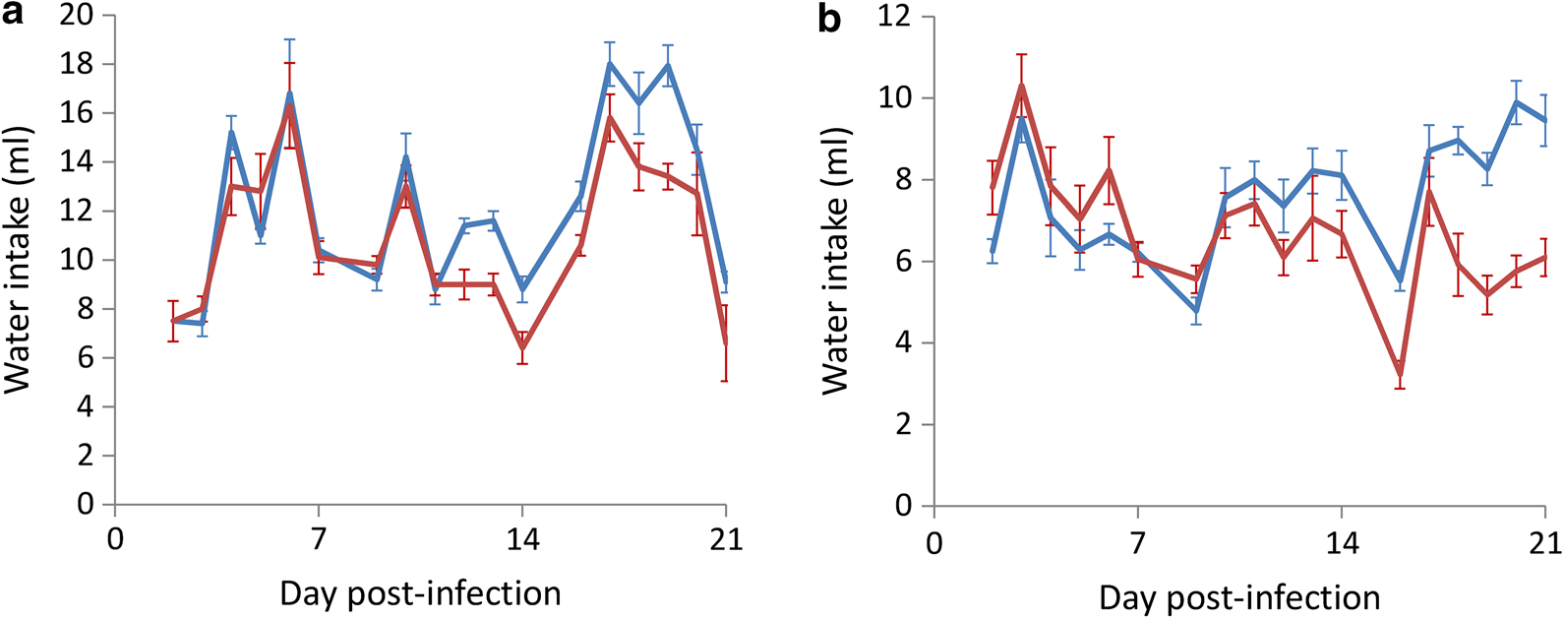
Effects of the Morris water maze test on the water intake in BALB/c (**a**) and C57BL/6 (**b**) mice infected with *Angiostrongylus cantonensis*. *Key*: blue, uninfected; red, infected

In BALB/c mice, there was no significant difference in water intake between the uninfected controls and infected mice in the swimming group up to day 11 post-infection (*t*_(9)_ = 0.794676, *P* > 0.05). From day 12, the water intake in the infected group significantly decreased (*t*_(9)_ = 0.002576, *P* < 0.01) (Fig. 5a). There was also no difference in food intake in C57BL/6 infected mice in the swimming group and the uninfected controls up to day 13 (*t*_(9)_ = 0.204049, *P* > 0.05); however there was a significant decrease of food intake in the infected mice from day 14 (*t*_(9)_ = 0.019749, *P* < 0.05) (Fig. 5b).

### Depression and anxiety-like behavior

**Fig. 6:**
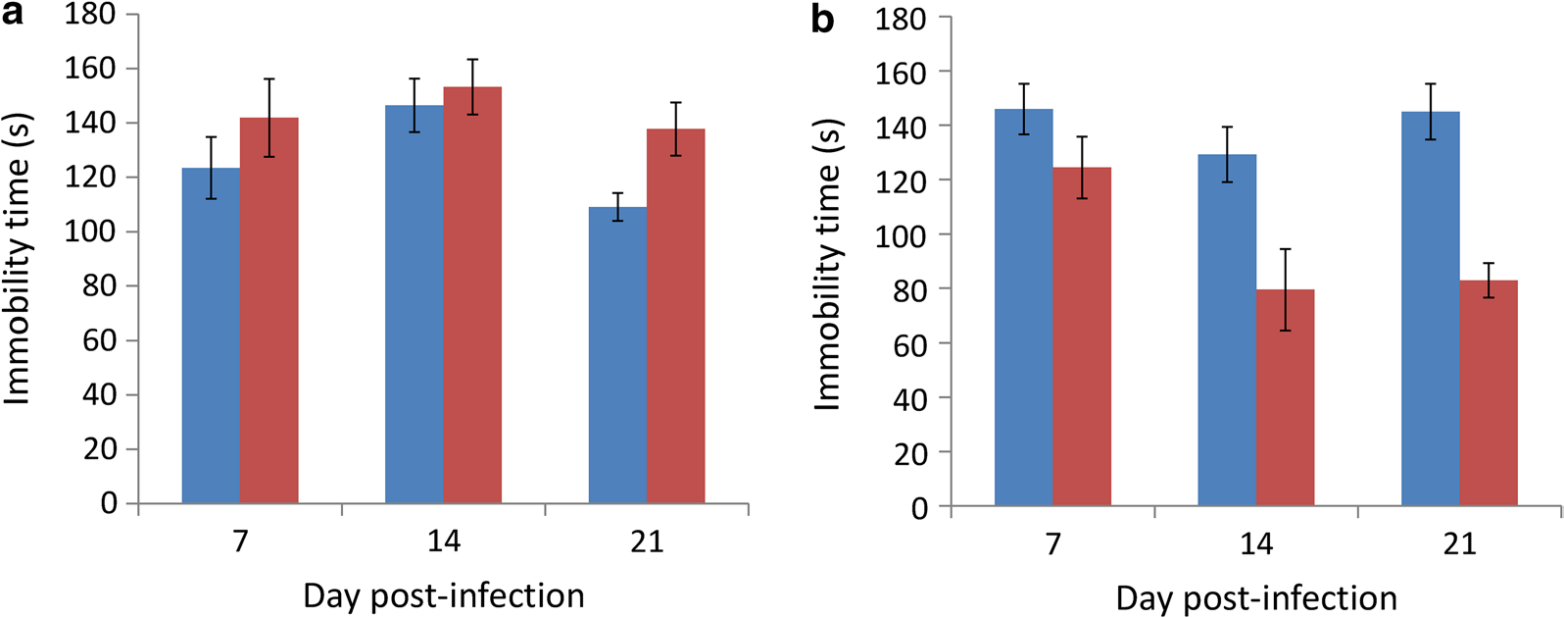
Determination of depression-like behaviors in BALB/c (**a**) and C57BL/6 (**b**) mice infected with *Angiostrongylus cantonensis* by the force swimming test. *Key*: blue, uninfected; red, infected

**Fig. 7:**
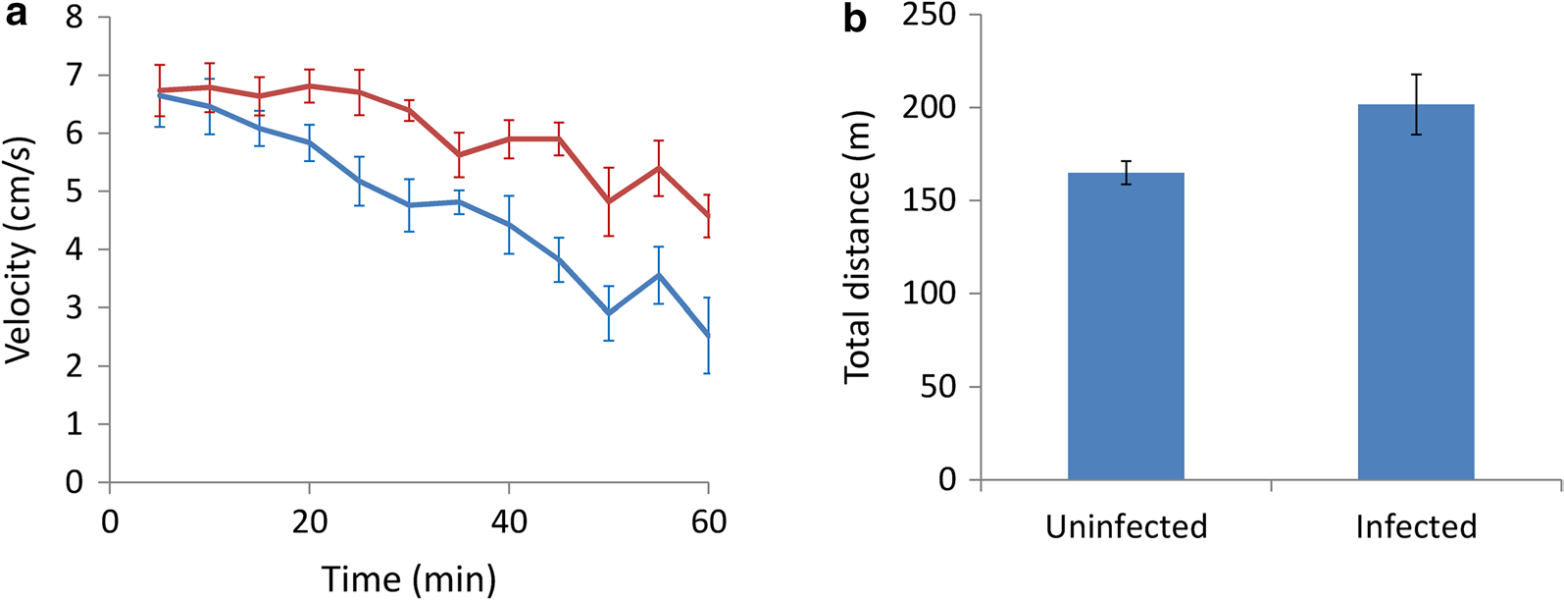
Anxiety-like behaviors in C57BL/6 mice infected with *Angiostrongylus cantonensis* determined by the open field test on day 9 post-infection. Activity (**a**) and walking distance (**b**). *Key*: blue, uninfected; red, infected

Using the force swimming test, on day 21 it was determined that infected BALB/c mice had a longer immobility time than the uninfected mice, indicating the occurrence of depression (*t*_(9)_ = 0.018582, *P* < 0.05) (Fig. 6a). No significant difference was found in immobility time in the infected and uninfected C57BL/6 mice on day 7 (*t*_(9)_ = 0.158094, *P* > 0.05). Moreover, the immobility time in the infected mice was found to be significantly shorter than the uninfected mice on day 14 (*t*_(9)_ = 0.013031, *P* < 0.05) and day 21 (*t*_(9)_ = 0.00011, *P* < 0.001), indicating an increase in activity and no occurrence of depression (Fig. 6b). Although no special findings were observed in the infected BALB/c mice by the open field test, the infected C57BL/6 mice were found to have significantly higher activity (*t*_(9)_ = 0.024914, *P* < 0.05) (Fig. 7a) and a longer walking distance (*t*_(9)_ = 0.029771, *P* < 0.05) (Fig. 7b) compared to the uninfected mice on day 9 post-infection, indicating that anxiety-like behavior occurred in these animals (Fig. 7).

### Changes in spatial learning

**Fig. 8:**
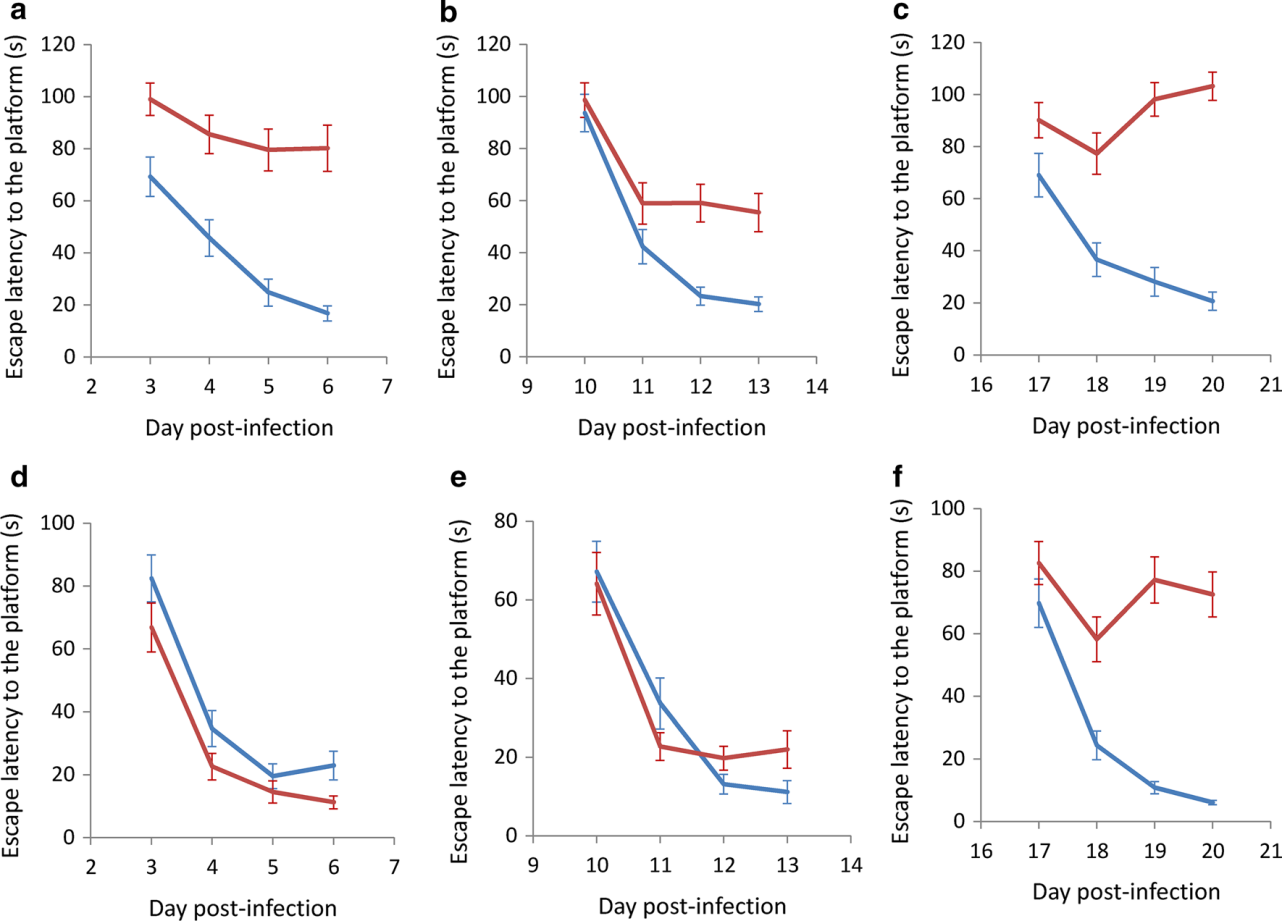
Comparison of spatial learning ability in mice by infected with *Angiostrongylus cantonensis* by determining escape latency to the platform in the Morris water maze test. **a**–**c** BALB/c. **d**–**f** C57BL/6 mice. *Key*: blue, uninfected; red, infected

After infection with *A*. *cantonensis*, BALB/c and C57BL/6 mice were continuously trained in the water maze for 4 days to observe their spatial learning ability. Before day 7, no significant difference in escape latency to the platform was found in infected and uninfected BALB/c mice (*t*_(9)_ = 0.561855, *P* > 0.05) (Fig. 8a). From day 12, the latency in the infected mice was found to be significantly higher than that of the uninfected group (*t*_(9)_ = 0.000049, *P* < 0.001) (Fig. 8b and c), indicating that the spatial learning ability of infected mice significantly decreased. Although infected and uninfected C57BL/6 mice showed no significant difference in spatial learning ability from day 1 to day 14 (*t*_(9)_ = 0.803257, *P* > 0.05) (Fig. 8d), the latency in the infected mice became significantly higher after day 14 (*t*_(9)_ = 0.013565, *P* < 0.05) (Fig. 8e, f), indicating a significantly lower spatial learning ability, this also occurred in the infected C57BL/6 mice, although the time in which it occurred was delayed.

### Changes in spatial memory

**Fig. 9:**
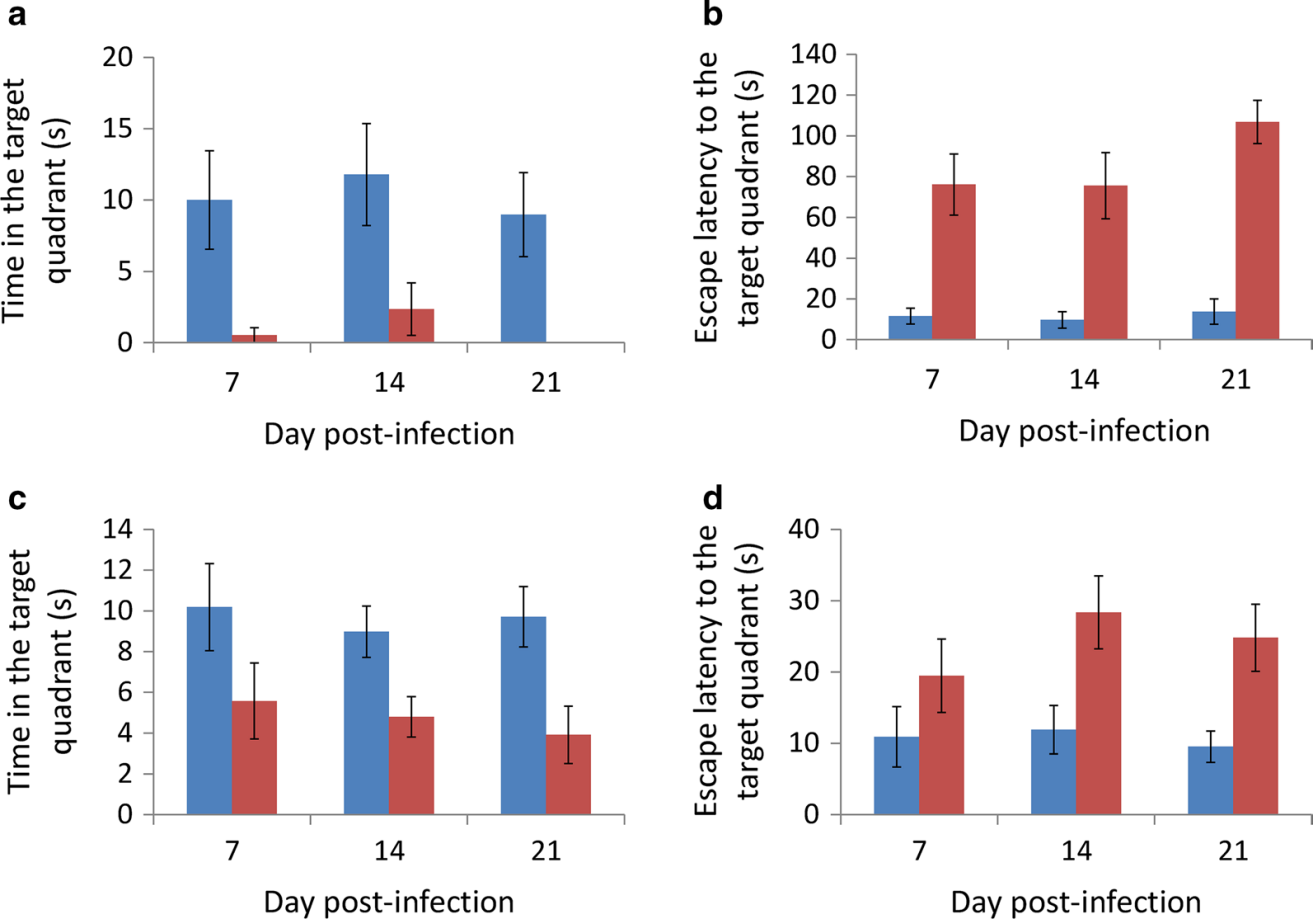
Comparison of spatial memory in mice infected with *Angiostrongylus cantonensis* by determining time in the target quadrant (BALB/c (**a**) and C57BL/6 (**c**)) and escape latency to the target quadrant in the Morris water maze test (BALB/c (**b**) and C57BL/6 (**d**)). *Key*: blue, uninfected; red, infected

After removing the water maze platform, infected BALB/c mice stayed in the target quadrant of the original platform for a significantly shorter time than the uninfected group (*t*_(9)_ = 0.002013, *P* < 0.01) (Fig. 9a) and the time for the infected mice to move to the target quadrant was significantly longer than that of the uninfected group (Fig. 9b) on days 7 (*t*_(9)_ = 0.016921, *P* < 0.05), 14 (*t*_(9)_ = 0.015133, *P* < 0.05), and 21 (*t*_(9)_ = 0.000047, *P* < 0.001). In the infected C57BL/6 mice, this phenomena only occurred on days 7 (*t*_(9)_ = 0.03045, *P* < 0.05) and 21 (*t*_(9)_ = 0.004776, *P* < 0.01) (Fig. 9c and d). Although the spatial memory ability of the two strains of mice significantly decreased, the ability remained intact in the C57BL/6 mice in the early stage of *A*. *cantonensis* infection.

## Discussion

Mice undertaking the water maze test were required to swim for four days in the training stage. The tested infected BALB/c mice may survive after day 28 and C57BL/6 mice had a high survival rate of 87% (Fig. 2). Moreover, the weight loss in those undertaking the test also became lower (Fig. 3). It has been reported that moderate physical exercise may promote metabolism, blood circulation, muscle growth and immunity, thereby preventing or delaying cardiovascular disease [27] and diabetes [28]. In addition to elevation of the proinflammatory cytokines IL1β, TNF-α and IL-6 [29], exercise may reduce C-reactive proteins in patients with coronary arteriosclerosis [30] and Type II T helper cytokines (IL-5 and IL-13) in asthmatic BALB/c mice [31]. These findings indicate that regular exercise may ease the clinical symptoms of mice infected with *A*. *cantonensis*. However, this suggestion requires further experiments.

In our previous study, eosinophilic meningitis was found to occur from day 12 post-infection in ICR mice infected with *A*. *cantonensis*, and pathologic changes were not limited to the meninges but also in the cerebral parenchyma especially in the hippocampus [13]. Hippocampal reticular injury has been associated with depression [18] and the length of depression is proportional to the volume of hippocampal gyrus loss [19]. In addition, the hypoxia-pituitary-adrenal (HPA) axis loss of function [30] or persistent inflammation [32] may also lead to depression. By using the forced swimming test, infected BALB/c mice were shown to have a longer immobility time than the infected C57BL/6 mice. These phenomena may be associated with the hippocampus and has been reported to be proportional to the mRNA expression of inducible nitric oxide synthase (iNos) [33].

In this study, we demonstrated that the recovery rate of worms in the two strains of mice infected with *A*. *cantonensis* decreased on day 21 (Fig. 1) and mortality of the animals also began to occur around this day (Fig. 2). It is possible that most of the young adults may be eliminated by the host immune system. The worms which survived around day 35 may continue to live. Moreover, the weight of infected mice began to decline from day 14 (Fig. 3). This finding may be associated with a decrease in food (Fig. 4) and water (Fig. 5) intake. As the parasite develops from L3 to young adults from day 14 [34], these worms may cause inflammation in the brain of the host, resulting in behavioral changes. These phenomena may explain the results of the water maze test. Although the infected mice are able to find the hidden platform from day 14, the time that they remain in this location becomes significantly shorter and the spatial learning and memory abilities significantly reduced.

By using the forced swimming test, we demonstrated that infected BALB/c mice developed depression on day 21. However, infected C57BL/6 mice had a significantly shorter immobility time (Fig. 6). The mice became more active on day nine post-infection and had a total moving distance of 46 m more than the non-infected mice, as demonstrated by the open field test (Fig. 7). These findings suggest that the young adults of *A*. *cantonensis* may cause mechanical damage to the brain of the host. The water maze test also demonstrated that the infected BALB/c mice required a longer time to find the hidden platform and had a shorter residence time at this position than their C57BL/6 counterpart (Fig. 9), showing the spatial memory ability of infected BALB/c mice was more severely reduced.

Changes in the immune response have been associated with spatial memory. It has been shown that SCID BALB/c mice have a lower spatial memory. However, their spatial memory and learning abilities was significantly improved after injection of T cells [35]. A water maze test demonstrated that IL-4-deficient C57BL/6 mice had low spatial memory learning ability [36]. Intracerebral microinjection of a mixture of IL-4 and IL-13 effectively restored spatial memory and learning abilities in C57BL/6 mice with overexpression of the amyloid-protein precursor 23 gene (AβPP23) (Alzheimer’s disease) through the increase of the IL-4 content in the hippocampus [37]. Injection of IL-4 into C-4BL/6 mice with cerebral ischemia has been found to be effective in improving the spatial memory and learning abilities [38]. These results suggest that T cells may secrete IL-4 to reduce the loss of spatial memory learning ability in an injured brain.

## Conclusions

In conclusion, *A*. *cantonensis* is able to cause cognitive impairments in heavily infected mice. The infection may cause depression, anxiety, and impairments in spatial learning and memory in heavily infected mice. Moreover, significantly higher severity was observed in the Th-2 dominant BALB/c mice.

## Data Availability

Data supporting the conclusions of this article are included within the article.

## Abbreviations

AβPP23: Amyloid-protein precursor 23
HPA: Hypoxia-pituitary-adrenal
iNos: Inducible nitric oxide synthase
L3: Third-stage larvae
SD: Sprague-Dawley
